# 40 Implementation of Universal Behavioral Health Screening on the Burn Unit

**DOI:** 10.1093/jbcr/iraf019.040

**Published:** 2025-04-01

**Authors:** Deanna Denman, Alicia Williams, Leopoldo Cancio

**Affiliations:** United States Army Institute of Surgical Research Burn Center; United States Army Institute of Surgical Research Burn Center; United States Army Institute of Surgical Research Burn Center

## Abstract

**Introduction:**

Psychosocial distress is common among burn survivors, and early detection is crucial for timely intervention and referrals. Many studies highlight the benefits of psychological support during hospitalization, yet numerous burn centers lack embedded behavioral health services. Additionally, the psychological well-being of non-burn patients (e.g., those with soft tissue infections or other skin conditions) treated in burn centers is understudied. Reliance on psychosocial screening during outpatient visits may miss early symptoms and critical opportunities for inpatient intervention. The ABA Burn Center Verification Program requires that “the burn center provides brief psychological screening/intervention” (Criterion 16.4, a level 1 criterion).

**Methods:**

We implemented universal psychological screening for all hospitalized patients in our burn center. Screenings were conducted by behavioral health technicians within 72 hours of admission or when patients were alert and oriented, with follow-up screenings weekly for those hospitalized beyond 10 days. The screening protocol included the Injured Trauma Survivor Screen (ITSS), and assessments for depression (PHQ-9), anxiety (GAD-7), suicidal ideation (CSSRS), and post-traumatic stress (PC-PTSD). Screenings were overseen by a licensed psychologist who facilitated follow-up for elevated scores.

**Results:**

Out of 193 patients screened (137 burned & 56 non-burned), 45% endorsed some psychological distress on the initial screening, and 46% reported persistent symptoms. Common concerns included anxiety (34%) and depression (34%). Acute stress symptoms were present in 39% of patients. Among burn patients, TBSA (median = 4.75%, IQR = 1.5-8.5%) was not correlated with behavioral health outcomes (p >.05). Mean anxiety levels were higher among non-burn patients compared to burn patients (p <.05), with a trend toward higher depression levels among non-burn patients (p =.09). The ITSS accurately predicted in-hospital symptoms of acute stress (p <.05) and mood disturbances (p <.01) across all patients.

**Conclusions:**

Weekly universal screening identifies the psychological needs of inpatients, with high rates of distress observed in both burn and non-burn patients treated in burn centers. Behavioral health services should be available for all patients in these settings. This project also supports the utility of the ITSS in predicting psychological distress in burn centers.

**Applicability of Research to Practice:**

Embedded psychological support enhances compliance with ABA standards for psychological screening in burn care allowing for early detection which may facilitate timely intervention and referrals.

**Funding for the Study:**

N/A

